# Mild Cognitive Impairment and Neurofeedback: A Randomized Controlled Trial

**DOI:** 10.3389/fnagi.2021.657646

**Published:** 2021-06-14

**Authors:** Yotam Lavy, Tzvi Dwolatzky, Zeev Kaplan, Jonathan Guez, Doron Todder

**Affiliations:** ^1^Ophtalmology Department, Soroka Medical Centre, Beersheba, Israel; ^2^Beer-Sheva Mental Health Center, Ministry of Health, Faculty of Health Sciences, Ben-Gurion University of the Negev, Beer Sheva, Israel; ^3^Geriatric Unit, Rambam Health Care Campus, Haifa, Israel; ^4^The Ruth and Bruce Rappaport Faculty of Medicine, Technion – Israel Institute of Technology, Haifa, Israel; ^5^Department of Psychology, Achva Academic College, Beer-Tuvia, Israel

**Keywords:** neurofeedback, memory, mild cognitive impairment, EEG, alpha rhythm, memory, Alzheimer's disease

## Abstract

**Background and Objectives:** Mild cognitive impairment (MCI) is often a precursor of dementia, and in particular of Alzheimer's Disease (AD) which is the most common cause of dementia. Individuals with amnestic MCI are several-fold more likely to develop AD than the general population. Therefore, MCI comprises a well-detectable, early stage time-point for therapeutic intervention and strategic prevention. Based on common electroencephalographical (EEG) pattern changes seen in individuals with MCI, we postulated that EEG-based neurofeedback could help improve the memory performance of patients with MCI. Memory performance is of particular importance in these patients, since memory decline is the most prominent symptom in most patients with MCI, and is the most predictive symptom for cognitive deterioration and the development of AD.

**Methods:** In order to improve the memory performance of patients with MCI we used a system of EEG-based neurofeedback in an attempt to reverse alterations of the EEG that are known to be common in patients with MCI. Our protocol comprised the provision of positive feedback in order to enhance the activity level of the upper alpha band. Participants were divided to two groups receiving either neurofeedback training to enhance the upper alpha frequency (Experimental group) or random feedbacks (Sham group)

**Results:** We witnessed a significant improvement in memory performance in subjects in the experimental group compared to those in the sham group. This improvement was maintained for at least 1 month.

**Conclusions:** Neurofeedback may be a promising and affordable novel approach for treating the decline in memory witnessed in patients with MCI.

## Introduction

*Mild cognitive impairment* (MCI) is an important health issue worldwide. It is characterized by a decline in cognitive abilities without affecting daily functions. Importantly, MCI is associated with an increased risk for developing Alzheimer's disease (AD) (Drago et al., 2011; Mufson et al., 2012). Longitudinal studies have found a conversion rate for MCI to AD of >25% over a period of two and a half years, which represents almost seven times the rate observed in the general population (Boyle et al., 2006; Brodaty et al., 2014). The Fifth Edition of the *Diagnostic and statistical manual of mental disorders* (DSM-5) has redefined MCI as Mild Neurocognitive Disorder, placing increasing emphasis on evaluating specific neurocognitive functions (American Psychiatric Association, 2013). There are as yet no proven efficacious pharmacological treatments for MCI (Karakaya et al., 2013; Kasper et al., 2020). Although the diagnosis of MCI is mainly clinical, specific findings have been demonstrated in such modalities as EEG and magnetic resonance imaging (MRI). (Jelic et al., 1997; Yin et al., 2013).

*Characteristic changes in the EEG of MCI patients* are seen particularly in the posterior regions of the brain (Huang et al., 2000; Jelic et al., 2000; Babiloni et al., 2011). These include a general slowing of the EEG, expressed by lower peak alpha frequency (PAF), lower alpha rhythm power and higher power in lower frequencies (delta and theta). PAF is defined as the peak in power within the alpha band, which in turn divides the alpha band into lower alpha and upper alpha. These particular EEG changes have been shown to correlate with poor cognitive performance (Klimesch, 1999), and gray matter atrophy. These also may serve as an indication that an older person will develop MCI or that a person with MCI will progress to AD (Jack et al., 1999, 2005; Karas et al., 2004). Specifically, theta power correlates negatively with neuropsychological performance in MCI (Cummins et al., 2008), while upper alpha power and PAF correlate positively with cognitive performance. Anatomically, these EEG changes correlate with atrophy of the thalamus, hippocampus and basal ganglia (Wolf et al., 2004; Moretti et al., 2012a,b). A recent comprehensive article regarding EEG findings in dementia and pre-clinical dementia perspective also included screening recommendations (Babiloni et al., 2020).

*Neurofeedback* is a re-emerging technique for non-invasive neuromodulation. Prominent research areas for neurofeedback include conditions such as epilepsy and attention deficit and hyperactivity disorder (Egner and Sterman, 2006; Strehl et al., 2006). Studies have also demonstrated the effects of neurofeedback on cognitive performance. Studied populations range from young to old and from healthy to cognitively affected individuals. Escolano et al. (2011) demonstrated an increase in the power of the upper alpha band in young individuals following neurofeedback training, with a significant enhancement of working memory in subjects compared to controls. Zoefel et al. (2011) showed a significant improvement in a mental rotation task following training aimed at increasing the upper alpha band. Angelakis and his group conducted a small (six participants) double blind randomized controlled study where older participants in the experimental group were trained to increase their PAF (Angelakis et al., 2007). Results suggested that the neurofeedback training protocol had a beneficial effect on some executive functions but had no clear effect on memory. Wang and Hsieh (2013) found that theta band power training of healthy older participants was successful with regard to EEG findings as well as in improving attention and working memory. In a study where participants diagnosed with either AD or vascular dementia were trained using quantitative EEG-guided neurofeedback (Surmeli et al., 2016), regardless of the dementia type, an average improvement of six points in the Mini Mental State Examination score was observed.

In a recent pilot observational non-controlled study we showed an improvement in memory performance after delivering 10 sessions of neurofeedback (Lavy et al., 2019). This was accompanied by an incremental increase in the PAF. Based on these encouraging results we decided to conduct a single-blinded sham-controlled study, with both short term (immediately at the end of the intervention) ant long term follow up (a month post intervention), in order to explore the possible beneficial effect of EEG-neurofeedback on the memory performance of subjects diagnosed with MCI.

## Methods

### Participants

A total of 30 subjects (13 women and 17 men, mean age = 71.93, SD = 8.51) with normal, or corrected to normal, color vision participated in the study. Participants were diagnosed with MCI at the Beer-Sheva Mental Health Center. The diagnosis was based on a clinical evaluation and cognitive assessment performed by either a geriatric specialist or a psychiatrist. Inclusion criteria included age >50 years and a diagnosis of MCI. Exclusion criteria included any active neurological pathology or an axis one disorder. The study was approved by the Ethics Committee (IRB) of the Soroka University Medical Center, and all subjects provided written informed consent for participation in the study. Three participants discontinued their participation in the study following cognitive evaluation and prior to the initiation of training. The remaining participants completed all the training sessions and cognitive evaluations. Table 1 presents the demographic data of the participants.

**Table 1:** Baseline demographic data of the study participants according to group.

	**Experimental**	**Sham**	***P*-value**
*N*	15	15	
Gender (Male)	46.15%	46.15%	
Age	70.23 (6.65)	74.15 ± 10.85	0.33
Years of education	14.08 (2.90)	15.92 ± 4.09	0.28

### Experimental Design

In this exploratory randomized controlled trial participants had a total of 12 encounters and were randomly assigned in a ratio 1:1 to either an experimental group or to a sham neurofeedback training group. In the experimental group participants were trained to increase the power of the individual upper alpha band at the central parietal location (EEG electrode - Pz). In the sham neurofeedback training, the feedback was given according to the power measurement from random electrodes (this will be explained in the next section) and the threshold for receiving feedback was arbitrarily re-determined every few minutes. To conform with the training conditions of the experimental group, the sham group received the same number of feedbacks per minute, and in a similar pattern to that of patients undergoing neurofeedback training.

The pre-training encounter was dedicated to cognitive evaluation using the NeuroTrax™ computerized cognitive assessment battery. This battery was validated in the MCI population (Dwolatzky et al., 2003; Lifshitz et al., 2012). Subsequent to the cognitive assessment, each participant participated in ten neurofeedback training sessions. Each session was structured to commence with the performance of a baseline resting EEG (3 min with eyes closed and 3 min with eyes open), followed by 30 min of neurofeedback training, and to conclude with a resting EEG. On completion of the ten training sessions, participants completed the NeuroTrax™ battery a second time, and again at 30 days after the last training session.

### EEG Recording

EEG was recorded using a Deymed Truescan 32 acquisition device. Each participant wore a 10/20 EEG cap suited to their head size. The EEG was recorded from 19 channels, at 256 samples per second with electrodes referenced to Fcz (according to the 10/20 international system). Each training session was divided to ten rounds in cycles of 3 min. In the experimental group the reward was given only according to the reading from the Pz electrode. In the sham group, in each round, the participant was given a reward according to the reading from different electrodes (Fpo1, Fpo2, F3, F4, Fz, C3, C4, Cz, T3, T4). Impedances were at 5 kΩ or less. For the analysis of the EEG, we used both WinEEG software and NeuroGuide software. The WinEEG software allows defining band ranges manually which is essential for calculating the peak alpha frequency and the power of the individual upper alpha. The NeuroGuide, however, allows the comparison of data to a normative database, allowing to extract a Z-score. Independent component analysis and visual examination were used to extract artifacts from the raw EEG. A rhythm pass filter was applied (0.50–30 Hz) to extract the relevant frequencies.

### Neurofeedback Protocol

For each participant an individual PAF with eyes closed was determined at the first encounter. Calculation of PAF was according to a baseline measurement with eyes closed before commencing the training session. Each participant was trained to increase the power of the EEG in the range of frequencies between his individually measured PAF and PAF + 2 Hz (for example if PAF = 8 Hz, the participant was trained to increase the power of alpha to the range of 8–10 Hz). Feedback was given according to a measurement from the Pz location in the midline, above the parietal lobe. We used the Deymed Truescan 32 acquisition device with a special case of the short-time Fourier transform, the Gabor transform. Training sessions were performed with eyes open and were divided into ten 3-min trials, each separated by a 10-s break. During training participants received positive feedbacks if they surpassed a pre-determined threshold of electrical activity (measured in uV) for at least 250 milliseconds, within the range of frequencies formerly described (PAF – PAF +2). The threshold value for gaining positive feedback was determined such that each participant would surpass the threshold eight times per minute at the beginning of training. The threshold was re-determined when a participant had achieved more than 30 feedbacks per minute. The feedbacks included a visual feedback of a continuous nature, composed of a three-dimensional game in which balls approached the middle of the screen as the participant approached the threshold, and a discrete auditory feedback presented as a beeping sound for each time that the predetermined threshold was surpassed. During each round, a counter was displayed on the screen showing the number of feedbacks a participant had gained since the beginning of the block. At the end of each block, the participant was shown a histogram of the scores for all the blocks from the beginning of the session and the overall number of feedbacks that the participant had received since the beginning of the training session. Participants were instructed to adopt any suitable strategy to achieve more feedbacks. In addition, it was explained to the participants that the movement of the balls and the beeping sounds give a live representation of brain activity in a certain area of the brain, and that every time they receive a feedback it means that they had surpassed a certain operator-determined threshold of brain activity that positively correlates with improved memory performance. For participants in the sham group, feedbacks were according to the activity of the random electrodes. In each round we used a different frequency band for the sham treatment (6–8, 12–14, 14–18 Hz). Therefore, the sham group were trained at different locations and frequency bands that were changed every 3 min. The process of randomization of feedback was done manually by the operator.

### Computerized Cognitive Testing

Computerized cognitive testing was performed using the NeuroTrax™ battery. During the evaluation, participants sat ~70 cm away from a computer screen. An experimenter supervised the participants throughout the tasks and provided clarification regarding instructions when needed.

The NeuroTrax™ battery (www.neurotrax.com) provides computerized cognitive testing. The tasks included in the battery used in our study were immediate and delayed verbal and non-verbal memory, Stroop interference, catch-game, Go-No-Go and visuospatial skills (Doniger, 2013).

## Results

Of the 27 subjects that underwent neurofeedback training, the cognitive findings of one subject were excluded from analysis. This was due to the fact that the subject had arrived at the post-training evaluation very stressed due to the death on that day of a close relative. His Cognitive performance at this evaluation was uncharacteristically poor. We used the EEG of the 9th session for the analysis of this subject.

### Cognitive Results

**Figure 1:**
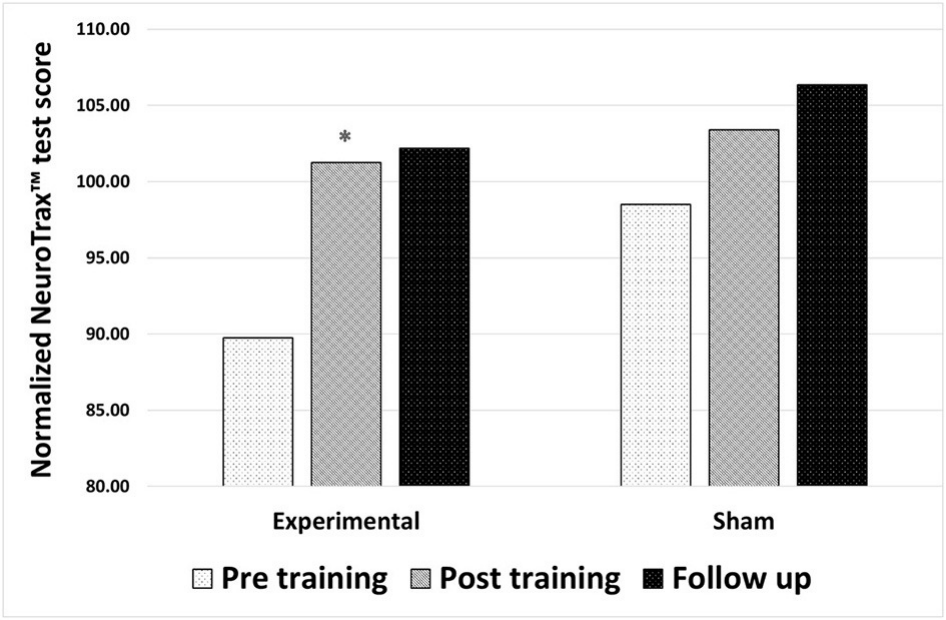
Changes in memory performance after either true neurofeedback training or sham neurofeedback training. Composite memory scores from the NeuroTrax™ tests in 3 time points: before training, after training and 30 days after training (follow up). Results of both the experimental and the sham neurofeedback groups are shown. A significant improvement in memory performance was observed for participants in the experimental group alone *P < 0.05.

*For participants in the experimental group*, statistical analysis revealed a significant improvement in the composite memory score on the NeuroTrax™ tests following neurofeedback training (*p* = 0.003; *Z* = −2.97). The baseline normalized average score of 89.76 ± 14.49 points increased to 101.25 ± 11.59 points (with 100 being the average in a normal, healthy population, matched for age and the level of education). Participants in the sham neurofeedback training group showed no statistically significant change in the composite memory score following neurofeedback training (*p* = 0.071; *Z* = −1.8), from an average normalized score of 98.5 ± 15.81 points at baseline to 103.39 ± 11.47 points following sham training. The improved memory performance of the experimental group did not significantly change from the post-training value to the follow-up evaluation (30 days post training) (*p* = 0.13; *Z* = −1.5), with an average score of 106.35 ± 9.16 points at follow-up (Figure 1).

For the other cognitive domains evaluated, results were as follows: Executive functions did not significantly change following training. For participants in the experimental group, value for pre, post and follow up were 99.81 ± 11.76, 103.28 ± 13.44 (*p* = 0.196; *Z* = −1.29) and 106.46 ± 18.21 points (pre to post: *p* = 0.86; *Z* = −0.18), respectively. For participants in the sham group, the baseline values for pre, post and follow up were 101.13 ± 10.23, 107.05 ± 10.46 (*p* = 0.071; *Z* = −1.8), and 106.65 ± 10.56 points (pre to post: *p* = 0.6; *Z* = −0.52), respectively.

In the evaluation of attention both groups seemingly improved following training. After correcting for multiple comparisons, however, these differences remained insignificant. Pre, post and follow up values for the experimental group were 100.79 ± 13.07, 103.2 ± 10.04 (*p* = 0.05; *Z* = −1.96) and 104.97 ± 9.7 points (*p* = 0.49; *Z* = −0.7), respectively. For the sham group, scores were 106.88 ± 3.29, 108.87 ± 4.13 points (*p* = 0.028; *Z* = −2.2) and 108.11 ± 7.53 points (*p* = 0.81; *Z* = −0.24), respectively.

The initial score in visuospatial ability in the experimental group was 98.06 ± 12.14 points and 103.86 ± 19.04 points (*p* = 0.05; *Z* = −1.96) following training. In the follow-up period, the score had insignificantly decreased to 98.1 ± 20.26 points (*p* = 0.17; *Z* = −1.37). In the sham neurofeedback training group, the baseline was 97.17 ± 11.41 points and increased following training to 108.28 ± 15.23 points (*p* = 0.011; *Z* = −2.56). Follow-up score was 109.7 ± 11.85 points (*p* = 0.88; *Z* = −0.15). After correcting for multiple comparisons there was not a significant change in either group.

In summarizing these findings there was a significant improvement in memory performance in the experimental group but not in the sham training group. There were no significant changes in the other cognitive domains tested in the experimental group, while an improvement in visuospatial ability was observed in the sham group. After correcting for multiple comparisons with the rigorous Bonferroni correction, the improvement in memory performance was the only outcome measure which remained statistically significant (*P* < 0.0041).

#### Specific Memory Tasks

In the NeuroTrax™ immediate and delayed verbal recall task, for *immediate verbal recall*, participants in the experimental group significantly improved (*p* = 0.003; *Z* = −2.97) from pre-training to post-training. The baseline score was 88.36 ± 16.85 points and increased following training to a score of 105.54 ± 10.87 points, and later 100.16 ± 15.95 points at 30-day follow-up, with no significant difference from the post-training period (*p* = 0.08; *Z* = −1.78). For participants in the sham group, there was no significant improvement in immediate verbal memory (*p* = 0.14; *Z* = −1.47), going from a value of 99.61 ± 16.4 points at baseline, to 103.92 ± 11.92 & 104.5 ± 12.38 points at post-training and at follow-up, respectively (*p* = 0.83; *Z* = −0.21).

In the *delayed verbal recall* task, there was a positive trend for the experimental group (*p* = 0.07; *Z* = −1.82), going from 90.98 ± 18.35 points to 100.12 ± 13.52 points from pre-training to post-training. Later, in the follow-up period, the score remained unchanged with a score of 100.84 ± 14.38 points (*p* = 0.721; *Z* = −0.36). For participants in the sham neurofeedback group, there was also a positive trend in this task (*p* = 0.06; *Z* = −1.89) from a value of 95.96 ± 18.37 points at baseline, to a value of 103.88 ± 10.05 points following training. At 30-days follow-up, the score was 105.47 ± 11.02 points, unchanged from the post-training score (*p* = 0.57; *Z* = −0.56).

In the NeuroTrax™ immediate and delayed non-verbal recall task, in the *immediate non-verbal recall task*, a significant increase in performance was found for the experimental group (*p* = 0.019; *Z* = −2.34), starting at 92.69 ± 21.88 points at baseline and going to 102.16 ± 16.79 points, after training. At follow-up, the improved performance was maintained with a score of 106.62 ± 17.47 points (*p* = 0.239; *Z* = −1.177). For participants in the sham neurofeedback training, the baseline value of the *immediate non-verbal recall* task was 98.99 ± 12.89 points and this insignificantly increased to 101.85 ± 13.46 points following training (*p* = 0.359; *Z* = −0.92). At the follow-up period, the average score increased to a value of 109.62 ± 11.92 points (*p* = 0.034; *Z* = −2.12), this, however, remained insignificant after correcting for multiple comparisons.

In the *delayed non-verbal recall task*, there was a statistically insignificant increase for the experimental group (*p* = 0.195; *Z* = −1.3), going from a value of 93.14 ± 19.47 points at baseline and to 97.46 ± 16.04 points following training. At the follow-up period there was another insignificant increase to a score of 101.1 ± 13.41 points (*p* = 0.241; *Z* = −1.17). For the sham group, there was no significant improvement (*p* = 0.31; *Z* = −1.02), going from a value of 98.81 ± 16.98 points at baseline, to a value of 103.21 ± 18.63 points after training. At follow-up, there was no significant change (*p* = 0.58; *Z* = −0.56) with a score of 105.72 ± 11.42 points.

These results show that subjects in the experimental group managed to significantly improve their performance in both immediate verbal memory and immediate non-verbal memory, but this was not the case for delayed verbal and delayed non-verbal memory. Participants in the sham group showed no significant improvement in any of these tasks following neurofeedback training. Correcting for multiple comparisons with the rigorous Bonferroni correction, the improvement in memory performance was most likely due to an improvement in immediate verbal memory (*P* < 00312).

### EEG Results

Our results showed insignificant changes in the absolute power of the upper alpha band in Pz location for both groups, following neurofeedback training. Participants in the experimental group had an average baseline alpha power value of 4.54 ± 5.64 uV^2^ that increased by 18% following training to a value of 5.37 ± 7.43 uV^2^. This was, however, statistically insignificant (*p* = 0.66; *Z* = −0.44). At 30 days follow-up, the average value of the absolute upper alpha had decreased by more than half to 2.04 ± 2.77 uV^2^, and this was statistically insignificant (*p* = 0.12; *Z* = −1.57). For participants in the sham neurofeedback group, the baseline value of the absolute upper alpha was of 1.65 ± 1.91 uV^2^ and had later decreased to 1.38 ± 1.53 uV^2^ (*p* = 0.94; *Z* = −0.7). In the follow-up period, the average alpha power value had gone up to 4.17 ± 5.5 uV^2^ (*p* = 0.12; *Z* = −1.57).

There was no significant effect of training on the relative power of the individually determined upper alpha band in neither the experimental nor the sham group. Participants in the experimental group had an average baseline relative alpha power value of 0.24 ± 0.17 % and of 0.24 ± 0.18 % after neurofeedback training (*p* = 0.3; *Z* = −1.04). At the follow-up evaluation, the value was 0.2 ± 0.17 % (*p* = 0.03; *Z* = −2.177), similar to the post neurofeedback training value.

For participants in the sham neurofeedback group the initial value of the individually determined upper alpha was of 0.18 ± 0.13 uV^2^, and 0.17 ± 0.13 uV^2^ after sham neurofeedback training (*p* = 0.81; *Z* = −0.25). In the follow up evaluation the value had remained statistically similar at 0.17 ± 0.14 uV^2^ (*p* = 0.75; *Z* = −0.31).

There were no significant changes in PAF following training. The experimental group had a baseline PAF value of 9.32 ± 0.71 Hz which reached a value of 9.36 ± 0.98 Hz (*p* = 0.97; *Z* = −0.04) post-training. In the follow-up period PAF reached a value of 9.31 ± 0.69 Hz, still unchanged from the previous evaluation (*p* = 0.86; *Z* = −0.178). For participants in the sham neurofeedback group the initial PAF value was 9.57 ± 1.33 Hz, and 9.69 ± 1.34 Hz after sham neurofeedback training (*p* = 0.68; *Z* = −0.42). In the follow-up period the PAF value was 9.5 ± 1.23 Hz, similar to the previous evaluation value (*p* = 0.68; *Z* = −0.41).

## Discussion

In this study we show an interesting finding of memory improvement following neurofeedback intervention that was retained for up to 30 days in subjects with MCI. The results of this study are in a continuum with a preliminary trial we performed in patients with MCI, using the same EEG-based neurofeedback protocol used in the current study. While we found a significant improvement in memory performance after neurofeedback training no similar improvement was found in other cognitive domains. This finding is important not only for the demonstration of the specificity of treatment but also to negate the possibility that the improved score in the memory performance resulted from an improvement in attention. Since it may be truthfully claimed that improving the attention span, which is one of the known results of neurofeedback, may also cause improved performance in memory testing. This, however, should also result in an improvement in other cognitive domains. Other possible factors influencing memory performance, such as relaxation, encouragement from the supervisor during testing, and other unknown factors, are unlikely since this was a controlled study and no similar improvement in memory was detected in the sham group. When evaluating the components of the composite memory score, we found that the main contributor to the change in memory was an improvement in immediate verbal and non-verbal memory rather than delayed memory.

Electroencephalographical evaluation failed to yield significant changes in peak alpha frequency and alpha spectral power. There was no significant improvement in upper alpha power or PAF. These results are in contrast with a previous trial we performed where there was an increase in PAF correlating with the number of training sessions (Lavy et al., 2019). One explanation could be an underpowered study design: when powering this study, we aimed at detecting changes in memory performance and not EEG changes. Also, it is not clear that training of the upper alpha should cause an increase in PAF, and in support of this contention other studies have also failed to show such changes despite positive cognitive results (Zoefel et al., 2011).

The current paper belongs to a relatively small number of papers that have described prolonged effects of neurofeedback training. One should note that this is the second study in which we demonstrate a lasting effect of neurofeedback training (for up to 30 days) and further supports the hypothesis for prolonged effects of neurofeedback training.

With regard to the use of sham intervention in neurofeedback research it should be noted that there is no single accepted method for shamming neurofeedback treatment and different studies use various modalities. Participants tend to notice if the feedbacks are administered in a completely random fashion. This could make participants aware to the fact that they are in the control group rather than in the experimental group. To avoid such a scenario, we chose the give genuine feedback in random locations and at varying thresholds. In this way the subjects receive real feedback for short periods of time but with no training effect.

The main limitation of this study is the difference in the baseline memory performance between the two groups in spite of the fact that patients were randomly assigned to groups. This is clearly related to the small sample size. A larger sample size would allow for better randomization and improved statistical power with regard to differences between groups. It should be noted however that the memory scores from the neurotrax program are corrected for age and education level and therefore the average should be regarded as absolute and comparison between the groups is less indicative, which makes this limitation more forgivable. In addition, even though the groups in these study had an equal ratio of male to female, we did not take in consideration inter-sexual variability that might exist in response to therapy, and this may be of relevance considering that gender differences in AD prevalence have been described (Babapour Mofrad and van der Flier, 2019).

## Conclusions

Our interesting findings should encourage further research regarding the value of neurofeedback training in patients with MCI. Larger studies with the possible addition of functional imaging may provide further insights into this fascinating field.

## Data Availability Statement

The raw data supporting the conclusions of this article will be made available by the authors, without undue reservation.

## Ethics Statement

The studies involving human participants were reviewed and approved by Soroka Medical Center IRB. The patients/participants provided their written informed consent to participate in this study.

## Author Contributions

YL designed the study, provided neurofeedback and sham training to the participants, performed the statistical analysis, and prepared the manuscript. TD designed the study, obtained ethics committee approval, recruited the subjects, and prepared and edited the manuscript. ZK designed the study and reviewed the manuscript. JG designed the study, advised regarding the cognitive aspects of the study, performed the statistical analysis, and reviewed the manuscript. DT designed the study, advised regarding the neurofeedback aspects of the study, recruited the subjects, performed the statistical analysis, and reviewed the manuscript. All authors contributed to the article and approved the submitted version.

## Conflict of Interest

DT is an advisor for “Bestbrain,” a startup in the field of neurofeedback. The remaining authors declare that the research was conducted in the absence of any commercial or financial relationships that could be construed as a potential conflict of interest.
